# Dengue burden in India: recent trends and importance of climatic parameters

**DOI:** 10.1038/emi.2017.57

**Published:** 2017-08-09

**Authors:** Srinivasa Rao Mutheneni, Andrew P Morse, Cyril Caminade, Suryanaryana Murty Upadhyayula

**Affiliations:** 1 Biology Division, CSIR-Indian Institute of Chemical Technology, Tarnaka, Hyderabad 500 007, Telangana, India; 2 Department of Geography and Planning, School of Environmental Sciences, University of Liverpool, Liverpool, Merseyside L69 7ZT, UK; 3 NIHR Health Protection Research Unit in Emerging and Zoonotic Infections, Liverpool, Merseyside L69 3GL, UK; 4 Department of Epidemiology and Population Health, Institute of Infection and Global Health, University of Liverpool, Liverpool, Merseyside L69 3GL, UK; 5 National Institute of Pharmaceutical Education and Research, Guwahati 781 032, Assam, India

**Keywords:** dengue, extrinsic incubation period (EIP), India, rainfall, temperature

## Abstract

For the past ten years, the number of dengue cases has gradually increased in India. Dengue is driven by complex interactions among host, vector and virus that are influenced by climatic factors. In the present study, we focused on the extrinsic incubation period (EIP) and its variability in different climatic zones of India. The EIP was calculated by using daily and monthly mean temperatures for the states of Punjab, Haryana, Gujarat, Rajasthan and Kerala. Among the studied states, a faster/low EIP in Kerala (8–15 days at 30.8 and 23.4 °C) and a generally slower/high EIP in Punjab (5.6–96.5 days at 35 and 0 °C) were simulated with daily temperatures. EIPs were calculated for different seasons, and Kerala showed the lowest EIP during the monsoon period. In addition, a significant association between dengue cases and precipitation was also observed. The results suggest that temperature is important in virus development in different climatic regions and may be useful in understanding spatio-temporal variations in dengue risk. Climate-based disease forecasting models in India should be refined and tailored for different climatic zones, instead of use of a standard model.

## INTRODUCTION

Dengue is a vector-borne disease that is a major public health threat globally. It is caused by the dengue virus (DENV, 1–4 serotypes), which is one of the most important arboviruses in tropical and subtropical regions.^1, 2^ Other arboviral diseases are present in India, including Japanese encephalitis, West Nile virus, chikungunya fever, Crimean-Congo hemorrhagic fever and Kyasanur forest disease. Since the mid-1990s, epidemics of dengue in India have become more frequent, especially in urban zones, and have quickly spread to new regions, such as Orissa, Arunachal Pradesh and Mizoram, where dengue was historically non-existent.^3^ The epidemiology of dengue in India was first reported in Madras (now Chennai) in 1780, and the first outbreak occurred in Calcutta (now Kolkata) in 1963; subsequent outbreaks have been reported in different parts of India.^4, 5^ Since 1956, four serotypes (one to four) of dengue virus have been reported in various parts of the country.^6^ The total number of dengue cases has significantly increased in India since 2001. In the early 2000s, dengue was endemic in a few southern (Maharashtra, Karnataka, Tamil Nadu and Pondicherry) and northern states (Delhi, Rajasthan, Haryana, Punjab and Chandigarh). It has recently spread to many states, including the union territories.^3^ In addition to the increased number of cases and disease severity, there has also been a major shift in the geographical range of the disease. Dengue had been restricted to urban areas, but it has now spread to rural regions.^7^ The expansion of dengue in India has been related to unplanned urbanization, changes in environmental factors, host–pathogen interactions and population immunological factors. Inadequate vector control measures have also created favorable conditions for dengue virus transmission and its mosquito vectors. Both *Aedes aegypti* and *Aedes albopictus* are the main competent vectors for dengue virus in India.^8^ The number of dengue cases has increased 30-fold globally over the past five decades.^9^ Dengue is endemic in more than 100 countries and causes an estimated 50 million infections annually.^10^ Nearly 3.97 billion people from 128 countries are at risk of infection.^11, 12^ Individuals infected with dengue exhibit a wide spectrum of clinical symptoms ranging from asymptomatic to severe clinical manifestations, such as dengue shock syndrome.^13^ The WHO regions of Southeast Asia (SEA) and the western Pacific represent ~75% of the current global burden of dengue.^14^ A dengue vaccine, Dengvaxia(R), has been registered in several countries. Dengvaxia(R) is a live attenuated tetravalent vaccine that is currently under evaluation in phase 3 clinical trials in Asia (Indonesia, Malaysia, Philippines, Thailand and Vietnam) and Latin America (Brazil, Colombia, Honduras, Mexico and Puerto Rico).^15^ The protective efficacy of Dengvaxia(R) against virologically confirmed dengue in the respective individual trials has been estimated to range between 50.2% and 76.6% for different ages and serotypes.^16^ Dengvaxia(R) has not yet been approved by the Ministry of Health and Family Welfare, Government of India, because more clinical trials are thought to be necessary in India.^17^ Similarly, Indian pharmaceutical companies are developing an indigenous dengue vaccine candidate that protects against all four strains in clinical manifestations.^18^

Dengue outbreaks in SEA were recorded as early as the late 1940s, and they have continued to the present, exhibiting a larger disease burden. Dengue has been hyperendemic for decades in SEA, which has the highest reported incidence^19^ and is one of the most important hotspots, with a series of epidemics occurring every 3–5 years.^20^ Approximately 87% of the total population in the SEA region is at risk of dengue.^21^ In India, compared with other Asian countries, dengue was not widespread before the 1990s.

Many studies have reported changing spatial patterns in dengue transmission. The reasons for such changes are related to several factors, ranging from the globalization of travel and trade, which favors the propagation of pathogens and vectors, to climatic changes or modified human behavior.^22, 23, 24^ In 2007, the Intergovernmental Panel on Climate Change cautioned that between 1.5 and 3.5 billion people worldwide will face the risk of dengue fever infection during the 2080s, owing to climate change.^25^ Temperature and precipitation are important climatic factors in mosquito population and disease transmission dynamics.^26^ Temperature influences the developmental rates, mortality and reproductive behavior of mosquitoes. Precipitation provides the water that serves as a habitat for larvae and pupae.^26, 27^ According to the Intergovernmental Panel on Climate Change, the global average temperature has increased by ~0.6 °C over the past 35 years, and the variation in precipitation has increased.^28^ Warm temperatures and high humidity favor increased longevity of the adult mosquitoes and shorten the viral incubation period within the vector and its blood-feeding intervals, thus leading to faster virus replication and increased transmission intensity.^29^ The association between weather and dengue varies across geographical locations and socio-environmental strata.^30, 31^

Precipitation is often required to create and maintain breeding sites and consequently has a strong influence on vector distribution. Dengue is endemic in Thailand and Latin American countries, where a positive association between dengue prevalence and rainfall has been reported.^32, 33^ Aguiar *et al.*^14^ have shown that the risk of dengue infection in Brazil is highly seasonal and increases primarily during the rainy season, when vector infestation reaches its peak.^14^ Similarly, studies have also reported that in wetter conditions, compared with drier conditions, mosquitoes expand their spatial range, thereby leading to increased risk of dengue infection.^34^ In contrast, dry conditions can also lead to epidemics in urban settings, because vulnerable people with little access to water resources tend to store water in unprotected reservoirs near their households. This water attracts *Ae. aegypti*, which is anthropophilic, thus further increasing the risk of transmission.^35, 36^ In South American countries, several studies have shown a significant relationship between the warm phase of El Niño Southern Oscillation and dengue outbreaks.^37, 38^

The climate of South Asia is highly influenced by the Asian monsoon. India receives 75% of its rainfall during the southwest monsoon period from June to September.^39^ Indian monsoon rainfall provides ample breeding habitats for *Ae. aegypti*, thus leading to high vector densities.^36^ For tropical zones, including India, dengue is highly seasonal, and very limited research has been conducted to estimate the influence of climatic factors on the burden of dengue.

The extrinsic incubation period (EIP) is the viral incubation period between the time when a mosquito draws a viremic blood meal and the time when that mosquito becomes infectious. Since the 1900s, the EIP has been recognized as an important factor in dengue transmission dynamics.^40^ Vector competence and horizontal transmission of dengue highly depend on the EIP.^41^ Many studies have revealed that temperature influences the EIP; at higher temperatures within a viable survival temperature range for the vector, the DENV replicates faster, and the EIP shortens and increases the chance of a high proportion of mosquitoes becoming infective during their life span.^42, 43^ The EIP is an important determinant of the temporal dynamics of DENV transmission.^44^

The EIP plays an important role in modulating the occurrence of dengue cases/outbreaks in a given region. Decreasing the incubation period by 5 days can lead to a threefold higher transmission rate of dengue, and raising the temperature from 17 to 30 °C increases dengue transmission fourfold.^45^ Higher temperatures may increase the amount of feeding within the gonotrophic cycle, given the smaller body size and enhanced metabolism resulting from higher temperatures.^46^ Rohani *et al.*^47^ have reported that the EIP decreases when the extrinsic incubation temperature increases from 9 days at 26 °C to 5 days at 30 °C. Most researchers have examined the EIP of dengue virus type 2, whereas Rohani *et al.*^47^ have also examined the EIP for dengue virus type 4.

The EIP is generally assumed to range between 8 and 12 days.^9, 48, 49^ Most dengue models use fixed values for the duration of the EIP, and very few experimental studies have been carried out in this direction.^50, 51, 52^

Climatologically, the Indian climate is distinct, including six climatic zones. To understand the EIP in these different climatic zones, five dengue-endemic states (Punjab, Haryana, Rajasthan, Gujarat and Kerala) were selected to characterize changes in the EIP by using daily and monthly mean temperatures. Similarly, this study also further assessed the effect of rainfall on dengue burden.

## MATERIALS AND METHODS

### Epidemiological data

The dengue surveillance system in India is a passive surveillance system, wherein dengue cases are diagnosed by public health care professionals at different levels, for example, in sub-centers (at the village level), primary health centers (intermediate structures) and community health centers (with more than 30 beds). These health centers report the number of confirmed laboratory dengue cases to the district medical officer, who then forwards the information to the state government. The disease surveillance system is carried out by the state government and the National Vector Borne Disease Control Program (NVBDCP). The NVBDCP reviews the dengue in different states of India and systematically maintains the case data. Periodic reviews and field visits are made by health officials to review the dengue incidence and record the data daily. The NVBDCP, of the Government of India, reviews these data and provides technical assistance, funding and commodities to the endemic states and union territories. In addition to the NVBDCP, an integrated disease surveillance program was also established by the Government of India in 1999, and it covers 600 districts in India.^53^

For the effective control of disease outbreaks, rapid and precise diagnosis of dengue is of paramount importance. In India, dengue is diagnosed primarily on the basis of clinical manifestations (such as high fever, headache, retro-orbital pain, myalgia, arthralgia, rash and hemorrhagic manifestations) and laboratory diagnosis.^54^ Dengue cases are confirmed in the laboratory by the MAC ELISA method on the basis of the detection of IgM antibodies.

### EIP model

The country-level EIP was calculated by using the monthly mean temperatures for 1998–2014 from NCEP2 reanalysis temperature data. Similarly, the daily EIP was calculated for each day of the year by using the daily mean NCEP2 temperature data, and the seasonal EIP was also calculated by using the daily mean temperature. We calculated the EIP at different temporal scales to obtain EIP ranges for different climatic zones of India.

To investigate the effect of temperature on the extrinsic incubation period of dengue virus in India, we used the model described by McLean *et al.*^42^ This model relates the EIP with temperature as a covariate.^42^ The EIP (*n*, in days) was estimated for each state on the basis of temperature (*T*, in °C), by using the following equation:


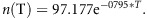


### Climate data

**Figure 1:**
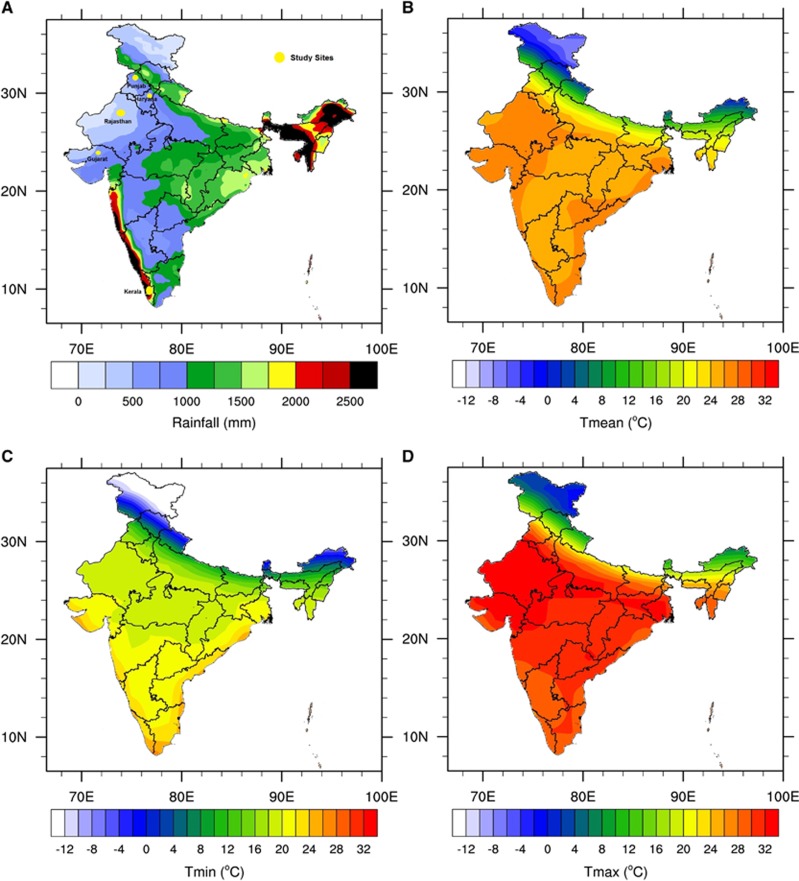
(**A**) Mean annual rainfall (mm) based on the TRMM satellite data and (**B**) mean (**C**) minimum and (**D**) maximum temperature (°C) based on the NCEP2 reanalysis data set. The annual mean is calculated for the 1998–2014 period. This figure was created using the National Centre for Atmospheric Research (NCAR) Command Language (NCL) version 6.1.2 (http://www.ncl.ucar.edu/).

Temperature data were derived by using the NCEP-DOE 2 reanalysis data set.^55^ This reanalysis data set uses a state-of-the-art analysis/forecast system to perform data assimilation on the basis of past observed data. This data set is available on a T62 global Gaussian grid (~1.8° × 1.8°) from 1979 to present, in daily time steps. Rainfall was derived from the tropical rainfall measuring mission (TRMM) data set. The TRMM data set is a satellite product that uses a ground rainfall station for calibration. The TRMM is available on a 0.25° × 0.25° spatial grid covering the tropics from 1998 to present at a daily time step. The TRMM was originally designed to improve observations of rainfall over the tropics.^56^ The annual means for rainfall and the maximum, minimum and mean temperature in India are shown in Figure 1. The average temperatures and rainfall for the five states of Haryana, Punjab, Rajasthan, Gujarat and Kerala are shown in Table 1.

### Statistical analysis

A bivariate Pearson correlation coefficient test was used to detect correlations between annual dengue cases and rainfall in Punjab, Haryana, Rajasthan, Gujarat and Kerala. The level of significance was considered to be 0.05. The correlation analysis was calculated by using the SPSS statistical software (version 22).

## RESULTS

### Epidemiological context of dengue in India

**Figure 2:**
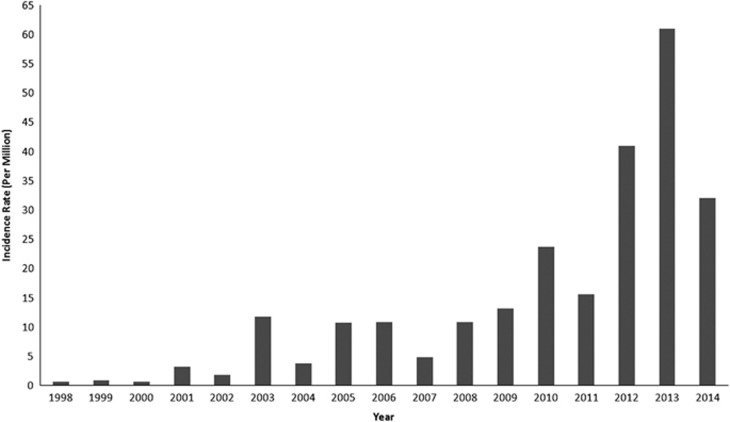
Dengue incidence rates (per million population) in India from 1998 to 2014. (Data source: NVBDCP, Govt. of India).

Since the 1990s, epidemics of dengue have become more frequent in many parts of India. Over the period 1998–2009, 82 327 dengue cases (incidence: 6.34 per million population) were reported. During a more recent period (2010–2014), 213 607 cases (incidence: 34.81 per million population) of dengue fever were observed. Thus, the number of dengue cases during the past 5 years has increased markedly, by a factor of ~2.6, with respect to the 1998–2009 period (Figure 2).

The 1996 dengue epidemic that occurred around Delhi and in Lucknow, Uttar Pradesh, subsequently spread all over the country, causing 16 000 cases and 545 deaths. The dengue incidence sharply increased from 1998 to 2001 from 0.72 to 3.21 per million population. In 2003, 2005, 2006, 2008 and 2009, the dengue incidence exceeded 10 per million population. Since 2010, a dengue incidence of greater than 15 per million population has been reported annually (Figure 2). From 2010 onward, the states of Assam, Bihar, Jharkhand, Orissa and Uttarakhand and some union territories including Andaman and Nicobar Islands, Dadra and Nagar Haveli, and Daman and Diu have become endemic for dengue. India experienced the highest dengue incidence in 2012 (about 41 per million population), 2013 (61 per million population) and 2014 (32 per million population).

**Figure 3:**
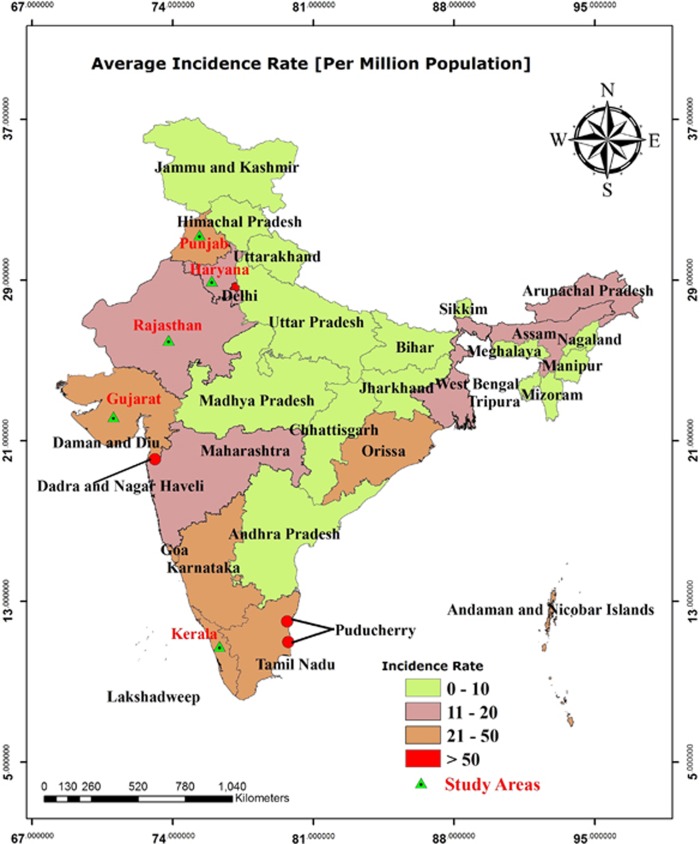
Average dengue incidence rates (per million population) by state in India from 1998 to 2014. The map was generated with ArcGIS-10.2.1 software (http://www.esri.in) from dengue case data.

From 1998 to 2014, the highest dengue incidence was reported in Pondicherry (372.92), followed by Dadra Nagar Haveli (176.31) and Delhi (102.15). Similarly, high dengue incidence, ranging between 21 and 50 per million, was reported for the states of Punjab, Gujarat, Karnataka, Kerala, Tamil Nadu and Orissa (Figure 3).

### Mean climatic conditions in India

The Indian monsoon, which usually starts in June and ends in September–October, brings rainfall from the Indian Ocean to the land. The highest level of precipitation occurs over the western coasts of India (states of Maharashtra, Goa, Karnataka and Kerala) and the far eastern states (Arunachal Pradesh, Assam, Manipur, Mizoram, Tripura and Meghalaya). More moderate rainfall is observed over the southeastern coasts (Tamil Nadu and Pondicherry), the eastern states and over the northern states bordering the Himalayas (Figure 1). Mean annual temperatures generally lie within the 20–30 °C range for most Indian states (Figure 1) except for the northern mountain states (Jammu & Kashmir, Himachal Pradesh and Uttaranchal), where more extreme cold winter conditions prevail.

### Temperature influence on dengue virus development within the mosquito vector

**Figure 4:**
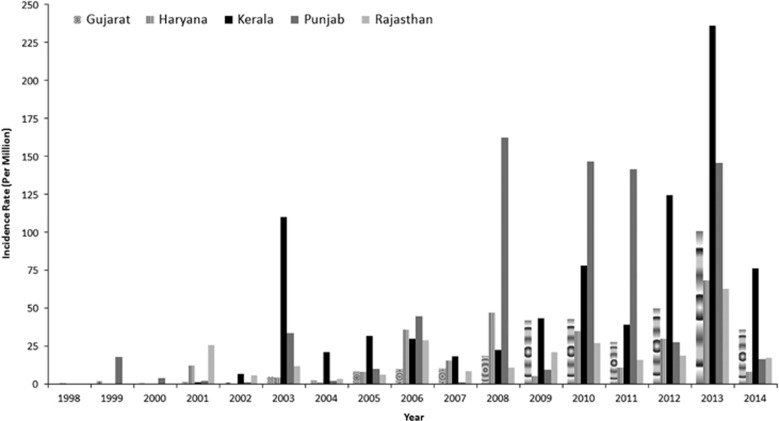
Yearly dengue incidence rates for different states of India. (Data source: NVBDCP, Govt. of India).

To understand the role of temperature on the development of dengue virus, five states in India were selected: Punjab, Haryana, Rajasthan, Gujarat and Kerala. These states were selected because they are very different in nature, both geographically and climatologically. Punjab and Haryana are humid, subtropical states, and they are located in northern India. Rajasthan and Gujarat states encompass an arid zone and are located in western India. The southern state of Kerala is located in a tropical wet and warm region. The mean annual temperatures for these five states, from the warmest to the coldest, were 27.3 °C (Kerala), 21 °C (Gujarat), 16.6 °C (Rajasthan), 14.8 °C (Haryana) and 10.1 °C (Punjab). Among these five states, the highest average dengue incidence from 1998 to 2014 was reported for the state of Kerala (49.27 per million population), followed by Punjab (44.89), Gujarat (21.04), Haryana (16.54) and Rajasthan (15.38). The state- and year-specific dengue cases for these five states are further shown in Figure 4.

**Figure 5:**
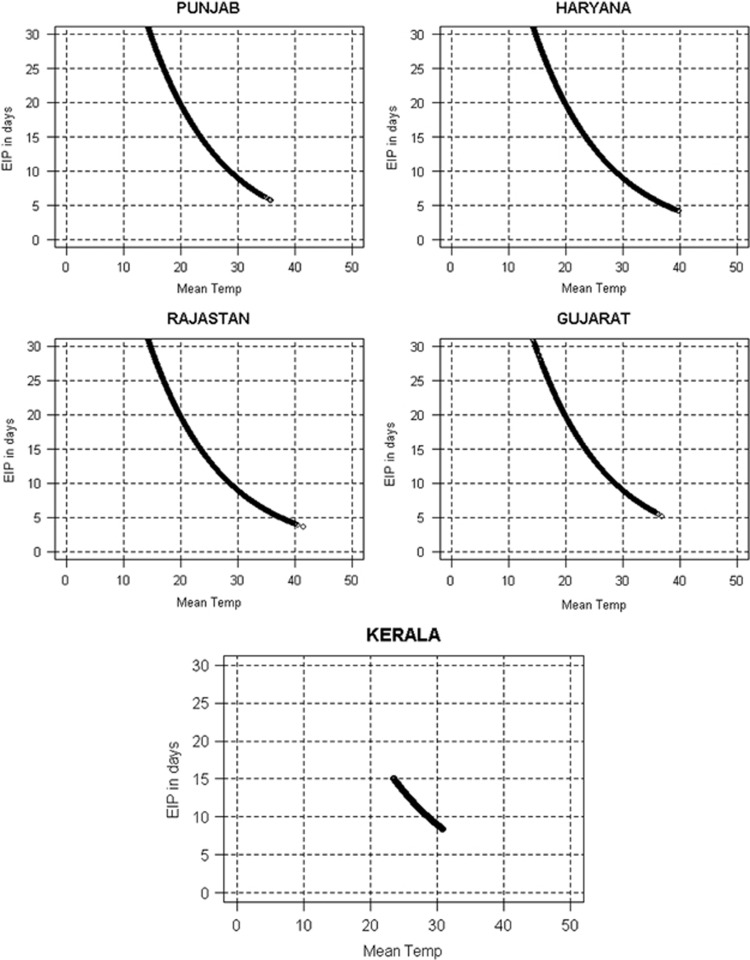
EIP (days) for DENV estimated from daily temperature data (from 1979 to 2014) for five states of India. The horizontal line (EIP=30 days) depicts a theoretical threshold, where EIP exceeds the maximum longevity of the mosquito vector.

The EIPs estimated by using daily mean temperature data for each studied state are shown in Figure 4. For the humid, subtropical state of Punjab, the virus development period was predicted to range between 5.6 (at 35 °C) and 96.4 (at 0 °C, see Figure 5) days. These simulated EIPs were compared with those of another subtropical and humid state, Haryana. The EIP in Haryana ranged between 4 (at 39.8 °C) and 80.7 (at 2.3 °C) days (Figure 5) on the basis of the daily mean temperature. These two states showed long EIPs, owing to the low temperatures reported after the monsoon period. The semi-arid Rajasthan state showed EIP values ranging from 3.6 (at 41.4 °C) to 85.3 (at 1.6 °C) days (Figure 5). Similarly, in Gujarat, the EIP ranged from 5 (36.8 °C) to 38.8 (11.5 °C) days on the basis of the daily temperature (Figure 5). The wettest and warmest state, Kerala, showed a narrow range of EIP values, varying from 8.3 (at 30.8 °C) to 15 (at 23.4 °C) days (Figure 5).

**Figure 6:**
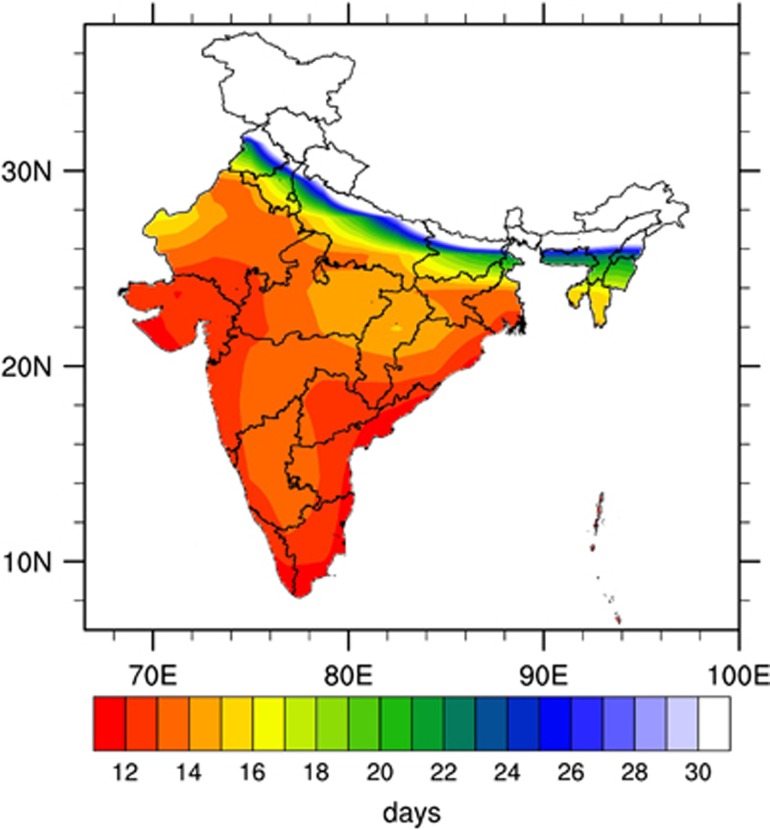
Mean annual EIP (in days) based on NCEP2 monthly mean temperatures calculated for the period 1998–2014. This figure was created using the NCAR Command Language (NCL) version 6.1.2 (http://www.ncl.ucar.edu/).

The mean annual EIP estimated by using monthly mean temperatures for India is shown in Figure 6. The eastern and western coasts of India showed the lowest EIP values, whereas the central, northern and northeastern parts showed higher EIP values. The northern and northeastern parts of India are highland areas and are located in the foothills of the Himalayas, where low temperatures are generally reported, thus leading to high EIP values. The lowest EIP values were mainly in the coastal states of India (Figure 6), a result relatively consistent with the dengue hotspots observed in Figure 3.

### Seasonal EIP prediction

In India, the climate is generally divided into four seasons: (a) the pre-monsoon season/summer period (March–May), (b) the monsoon period (June–August), (c) the post-monsoon period (September–November) and (d) winter (December–February). The temperature varies substantially across seasons, thus affecting EIP dynamics. The EIP was calculated for different seasons by using daily temperatures. The EIPs for the studied states during different seasons are shown in Table 2 and Supplementary Figure S1. Among all of the states, Kerala showed the lowest EIP (8–12.5 days) during the pre-monsoon period, followed by a similar range of EIPs observed in the winter, monsoon and post-monsoon periods (9–14.9 days). Gujarat showed low EIPs (5–13.5 days) during the monsoon period and again nearly similar pre-monsoon and post-monsoon EIPs (5.83–25.4, see Supplementary Figure S1).

In Punjab, the mean (±95% CI) EIP estimated using daily temperature was 24.48±0.24 days at 19.4 °C, Haryana: 16.98±0.17 days at 23.9 °C, Rajasthan: 14.28±0.15 days at 26.3 °C and Kerala: 11.07±0.01 days at 27.3 °C. Similarly, the mean (±95% CI) EIPs for different seasons predicted from daily temperatures are highlighted in Table 2, which indicates that the risk of dengue is particularly high during the monsoon period, when the EIP appears to be at a minimum in most states.

The correlation coefficients between annual dengue incidence and annual EIP at the state level showed low values (see Supplementary Table 1), thus indicating poor model performance in reproducing the observed annual dengue burden from year to year. This poor performance may be due to many reasons (for example, changes in surveillance across different states, or the virus still not being introduced in a given state). In contrast, the mean dengue incidence and simulated EIP showed significant negative spatial correlation (*r*=−0.33; *P*=0.94). This result indicates that the EIP model based only on temperature is able to discriminate among states with low/high dengue burden.

### Precipitation and dengue cases

To understand the role of rainfall on dengue transmission, annual rainfall data were extracted for the dengue-endemic states of Punjab, Haryana, Rajasthan, Gujarat and Kerala. Pearson correlation analysis showed a moderate to strong positive association between dengue cases and total precipitation, as well as rainy days greater than 1 mm or greater than 10 mm. The states of Punjab, Haryana, Rajasthan and Kerala showed significant associations between dengue cases and annual rainfall, as well as rainy days greater than 1 mm and greater than 10 mm, whereas the arid state of Gujarat did not show any significant association between rainfall and dengue cases (Table 3).

## DISCUSSION

Dengue is a major public health problem in India. Some studies have reported that an epidemiological shift in dengue viruses and climate change might be responsible for the observed increase in dengue burden across India.^57, 58^ Studies have focused on several epidemiological and entomological aspects of dengue and to a lesser extent on understanding the relevance of climatic factors, but none have investigated the extrinsic incubation period of the dengue virus within the mosquito vector. This is the first study to estimate the extrinsic incubation period by using temperature data for different states of India. The EIP plays a major role in dengue-endemic regions, where the vectors ingest the virus through a blood meal, and the virus escapes the mid gut, passes through the mosquito body and finally reaches the salivary glands and can be transmitted to another susceptible host. Most modeling studies have considered static EIP values rather than dynamic EIP estimates for a particular region.^59^ In this study, instead of using fixed values, we estimated EIPs by using daily mean temperatures for dengue-endemic Indian states and examined the association with the overall dengue burden at the country level and for five dengue-endemic states in India. This study also investigated EIPs for different seasons to understand dengue virus transmission dynamics.

The Indian climate is highly influenced by the Indian monsoon system. During the boreal summer, southwesterly winds bring moisture from the Indian Ocean to the land, thus resulting in heavy rains across India during the southwest monsoon period. This is followed by the northeast monsoon. Recent studies have shown that seasonal mean temperature in India has increased significantly over the past 100 years, with an increase of 0.9 °C during the post-monsoon period and 1.1 °C during winter.^60^ Slight increases in temperature can increase the dengue risk by increasing the mosquito development rate and shortening the virus incubation time, thereby increasing the rate of transmission. In India, temperature varies in different climatic zones at both temporal and spatial scales, and these variations influence the EIP. This influence is very pronounced in Punjab, Haryana and Rajasthan, where the EIP generally exceeds the average life span (45–49 days) of both *Ae. aegypti* and *Ae. albopictus* mosquitoes, even during intense dengue transmission periods (Punjab only).^61, 62^

Some studies have reported that daily temperature variation may play a major role in dengue virus transmission and vector–pathogen interactions.^63, 64^ Similarly, understanding of ectotherm ecology has improved, thus providing novel ideas on how to quantify the impact of anthropogenic climate change on pest and disease risk.^65, 66^ In general, the EIP for dengue ranges between 8 and 12 days.^9^ Chan and Johansson^44^ have calculated the mean EIP for a village in Taiwan, finding EIP values of 15 days at 25 °C and 6.5 days at 30 °C, whereas our study predicted 13 days at 25 °C and 9 days at 30 °C.^44^ Most studied states showed low EIP values during the monsoon period, whereas other seasons had large spatial variation in the EIP (Table 2). The northwestern parts of India usually exhibited low-temperature conditions during the late post-monsoon or winter periods, owing to cold conditions. In cool temperature settings, DENV cannot reproduce in mosquitoes, and transmission does not occur. An experimental study has also shown that below 18 °C, the virus cannot be found in the vector’s salivary glands, whereas at 21 °C, the viral antigen is detectable in *Ae. albopictus*.^67^ Similarly, above 20 °C, the dengue incidence gradually increases and peaks at ~32 °C before declining at higher temperatures.^68^

The EIP for dengue viruses has been found to decrease when the temperature increases from 26 to 30 °C, results similar to our findings.^47^ In tropical countries, including India, these temperatures are generally experienced during monsoon or early post-monsoon periods. Among the five states studied, Kerala experiences the highest number of dengue cases, possibly because of the availability of breeding grounds, a higher percentage of infected mosquitoes, suitable temperature ranges (23.5–30 °C) and subsequent short incubation periods in all seasons (9–14 days) and during the rainy season. These temperature ranges are mostly suitable for mosquito development and virus transmission. Similar studies have also reported a high prevalence of dengue in Mexico during the rainy season, when temperatures typically range between 17 and 30 °C.^45^

Temperatures in the lower range of dengue distribution (~17–18 °C) limits disease transmission through its effect on the EIP. High temperatures (~35 °C, depending on the vector species) tend to decrease disease risk, because they can limit mosquito survival. Consequently, future climate change might further affect dengue burden and that of other vector-borne diseases in India. In cooler areas, where temperature is a limiting factor, a slight increase in temperature might lead to disease transmission. As an example, dengue virus and its vectors have rapidly expanded their range into Himalayan countries, such as Nepal and Bhutan, as well as into northern states of India, such as Darjeeling, over the past 10 years.^69^

A recent study has compiled all dengue outbreaks in India^3^, showing that most dengue outbreaks occurred in Punjab, Haryana, Rajasthan, Gujarat and Kerala states during the monsoon or post-monsoon period. Thus, all study states are influenced by strong seasonality, underscoring the roles of both rainfall and ambient temperature in the potential transmission of dengue virus during monsoon and post-monsoon periods. Further studies are required to develop seasonal forecasting of dengue incidence in India.

During the past few decades, *Aedes* vectors have expanded their geographical range. Apart from dengue, *Aedes* vectors can also transmit other arboviruses, such as chikungunya and Zika virus.^70^ Chikungunya is already widespread in many countries, including India, whereas Zika virus is now an emerging arbovirus, and it has a similar epidemiology and transmission cycle to that of dengue virus in the tropical world.^71^ This study had some limitations, including the need to understand the incubation period according to serotype and by mosquito species. Our study highlights the association between weather and the EIP for different states of India, and we show that it is very difficult to develop a general model for the entire country. Hence, future studies should focus on the development of forecasting models by climatic zone and season. Other important parameters, including socioeconomic and demographic factors such as population density and migration, should be included in future risk assessment studies to further understand this complex and fast-growing disease.

## Figures and Tables

**Table 1:** Average dengue incidence rate (per million population) during 1998–2014 and average temperature (minimum and maximum) and rainfall calculated for the period 1961–1990 for five Indian states

**State name**	**Dengue incidence (per million population)**	**Mean minimum temperature (°C)**	**Mean maximum temperature (°C)**	**Average annual rainfall (mm)**
Haryana	16.544	13.8	33.4	635
Punjab	44.894	12.9	33.6	663
Rajasthan	15.38	15.5	33.3	478
Gujarat	21.045	19.6	32.1	688
Kerala	49.278	25	28.3	2375

**Table 2:** EIP days range and mean EIP days with ±95% CI for different seasons predicted using daily temperature data for the period 1979–2014

**States**	**Winter**	**Pre-monsoon**	**Monsoon**	**Post-monsoon**
Punjab	25.72–74.63 (44.38±0.33)	7.09–84.42 (20.18±0.31)	5.80–22.76 (11.32±0.09)	9.03–96.48 (22.43±0.29)
Haryana	14.34–80.75 (30.52±0.23)	5.05–48.12 (12.89±0.19)	4.08–14.68 (8.38±0.07)	7.02–40.50 (16.39±0.2)
Rajasthan	10.63–85.37 (26.76±0.24)	4.33–40.66 (9.97±0.15)	3.61–12.61 (7.29±0.06)	5.32–33.33 (13.33±0.18)
Gujarat	10.34–38.80 (18.58±0.12)	5.83–25.44 (9.66±0.08)	5.17–13.52 (9.44±0.05)	6.44–23.12 (11.33±0.08)
Kerala	9.61–14.26 (11.65±0.02)	8.61–12.50 (9.87±0.02)	9.16–14.92 (11.49±0.03)	9.61–14.20 (11.27±0.02)

**Table 3:** Pearson correlation analysis between annual dengue cases and annual rainfall

**Climate factors**	**Dengue cases**				
	**Punjab**	**Haryana**	**Rajasthan**	**Gujarat**	**Kerala**
Dengue cases	1	1	1	1	1
Rainy days TRMM >1 mm	0.618*	0.546**	0.670*	0.458	0.352*
Rainy days TRMM >10 mm	0.735*	0.551**	0.620*	0.327	0.397*
Annual rainfall TRMM	0.775*	0.532**	0.604**	0.260	0.409*

* and ** denote significant correlations at the 0.05 and 0.01 significance levels, respectively.
