# Supplementary material for: Dengue burden in India: recent trends and importance of climatic parameters
Source: Emerg Microbes Infect. 2017 Aug 9;6(8):e70–. doi: 10.1038/emi.2017.57 (PMC5583666; doi:10.1038/emi.2017.57)

**Title:** Dengue burden in India: Recent trends and importance of climatic parameters

**Authors:** Srinivasa Rao Mutheneni, Andy Morse, Cyril Caminade, Suryanaryana Murty Upadhyayula

**Supplementary Figure S1:** EIP of dengue virus for different seasons calculated for the states of Punjab, Haryana, Rajasthan, Gujarat and Kerala using daily temperature data for the period 1979-2014 .

**Punjab seasonal EIP days:**

**
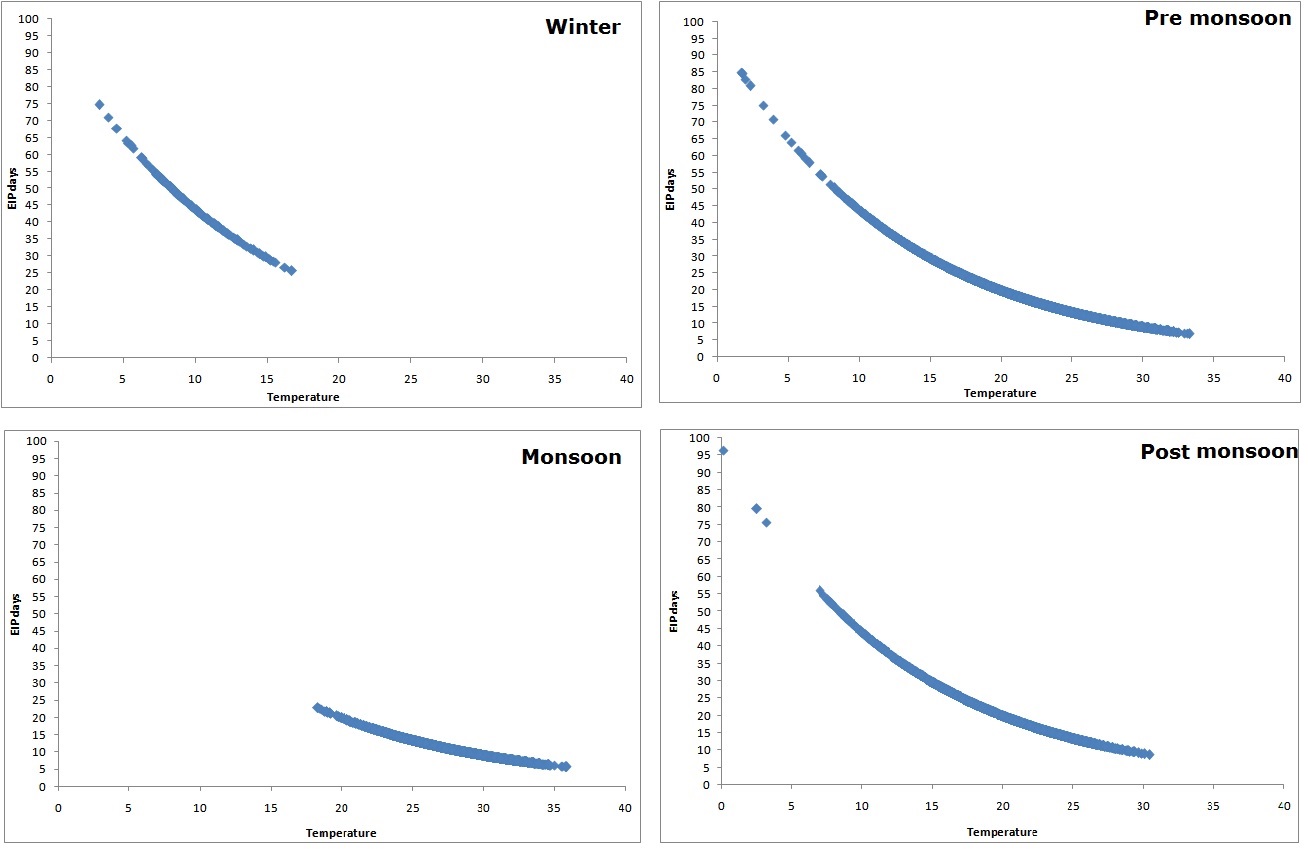
**

**Haryana seasonal EIP days:**

**
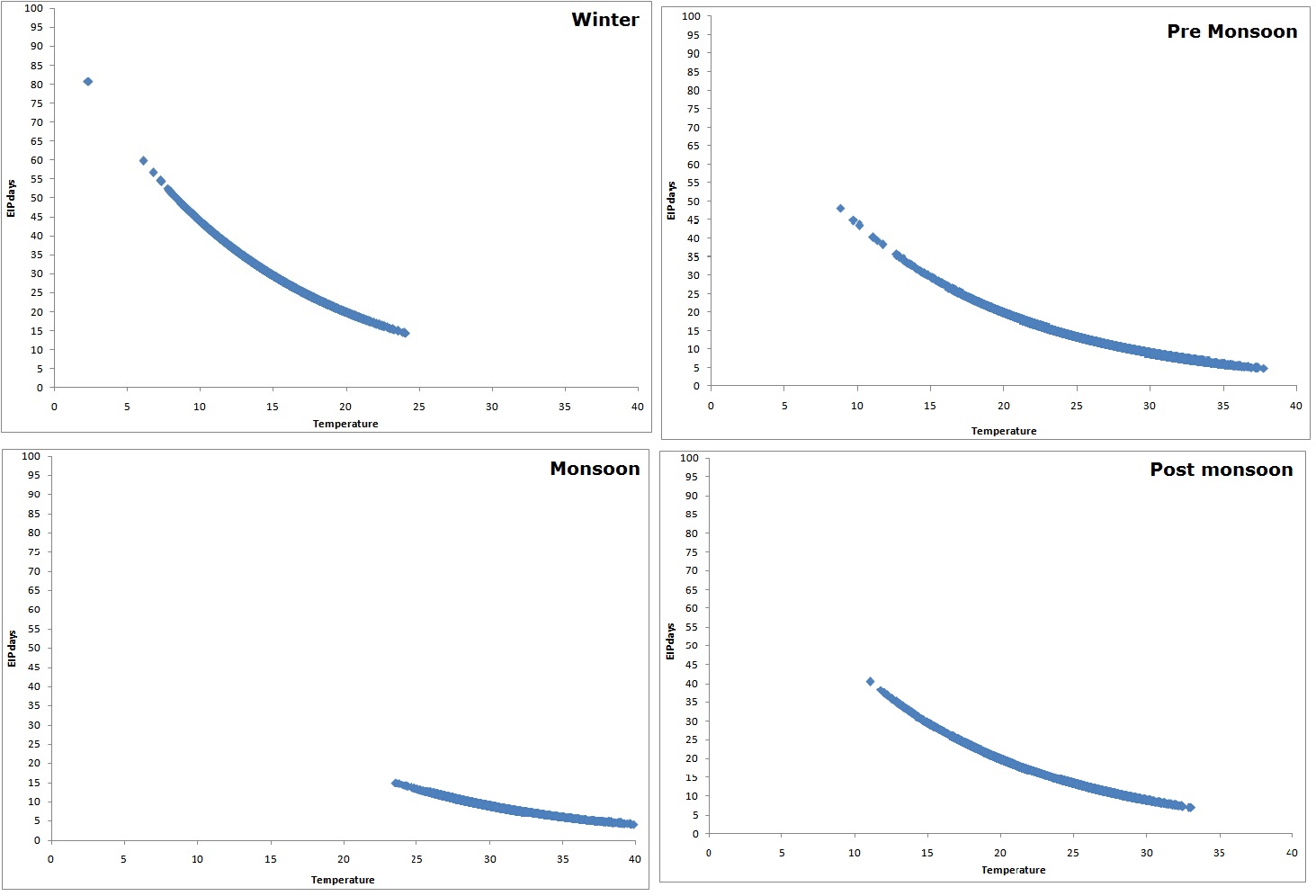
**

**Rajasthan seasonal EIP days:**

**
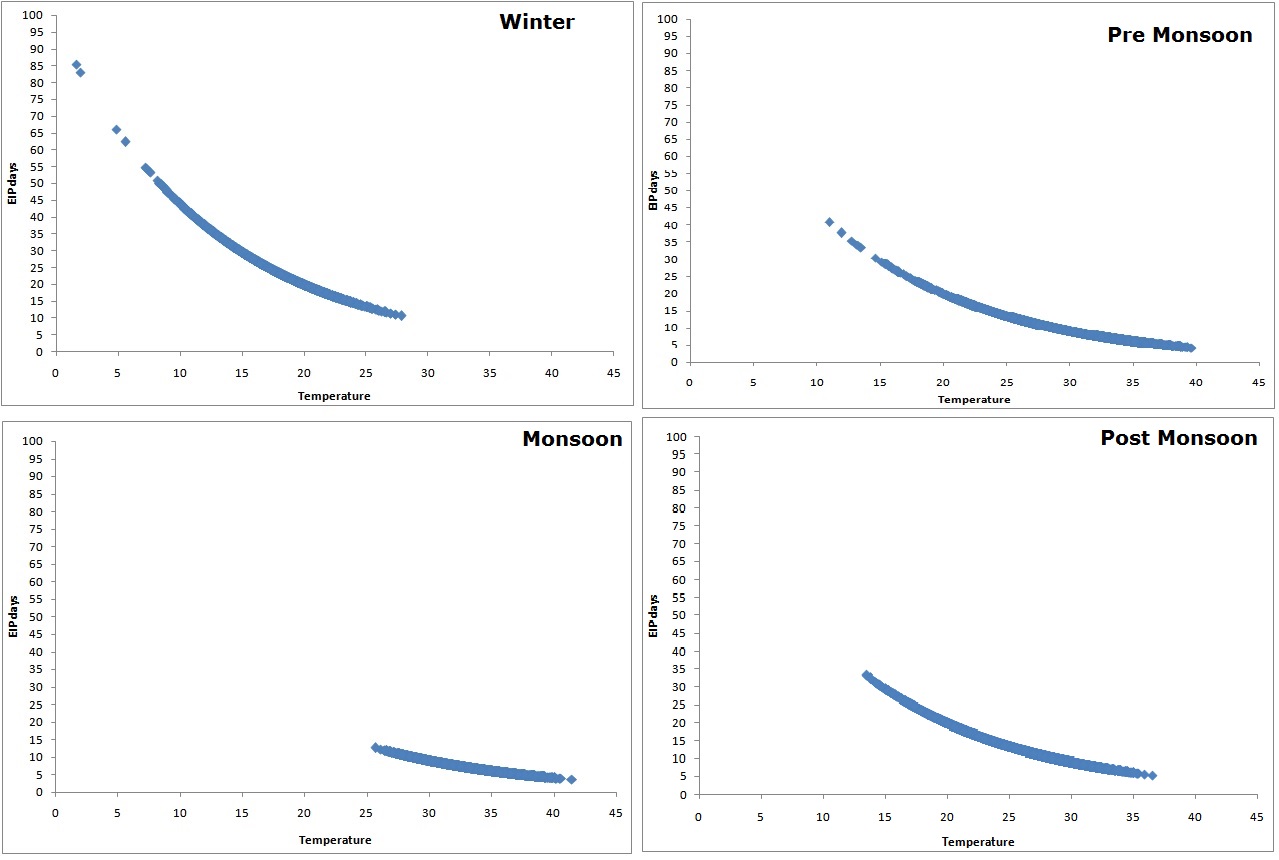
**

**Gujarat seasonal EIP days:**

**
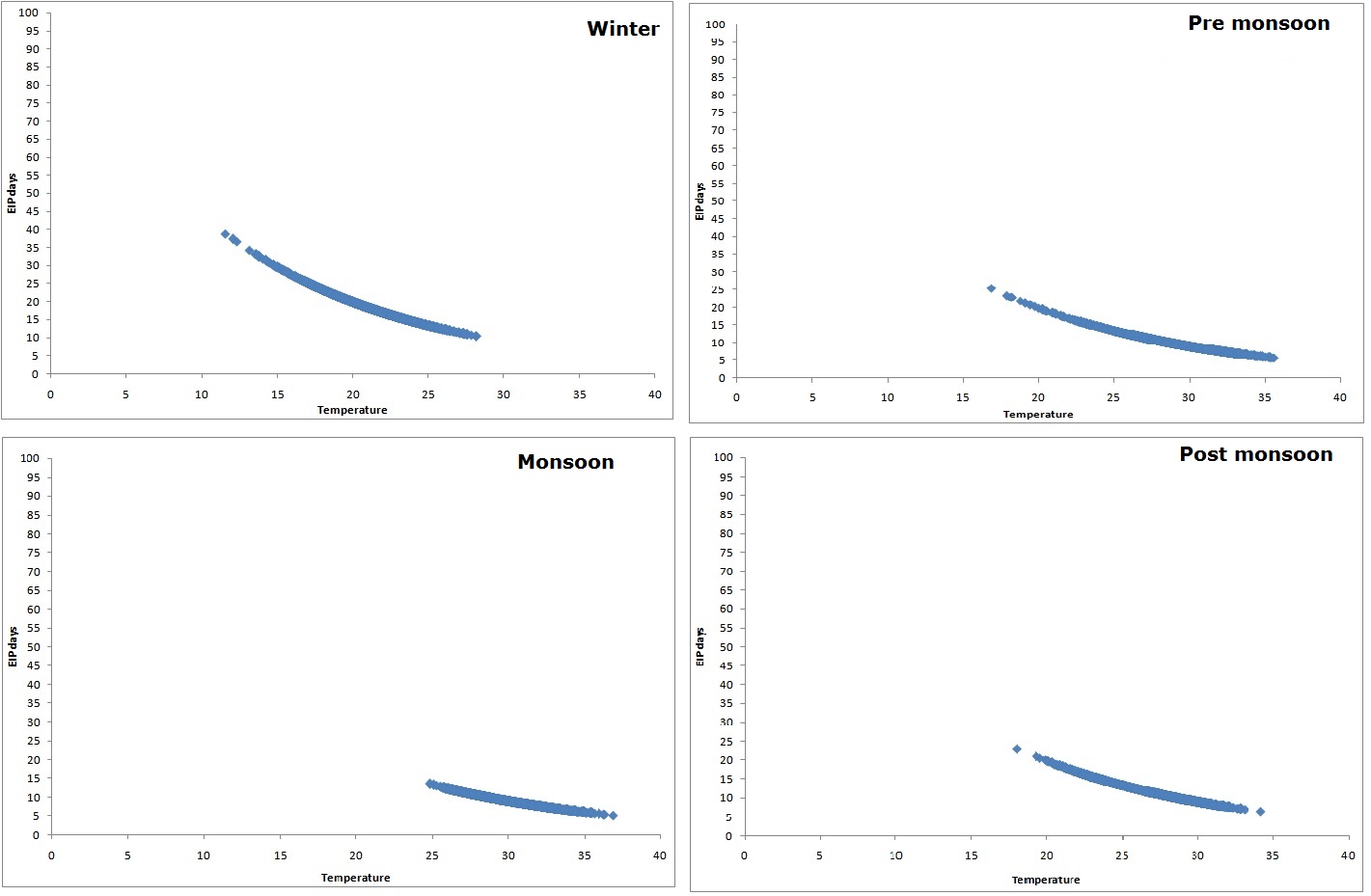
**

**Kerala seasonal EIP days:**


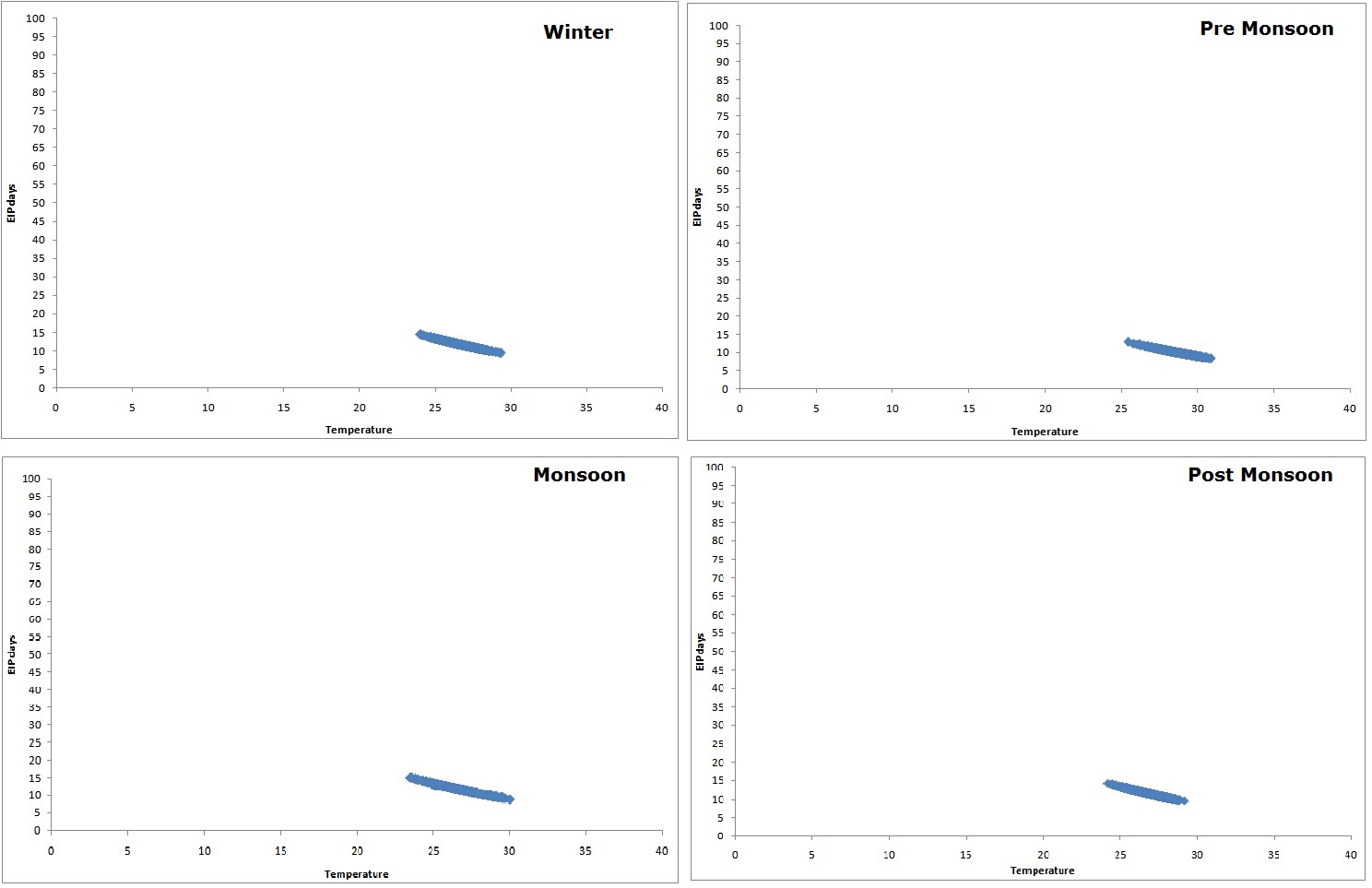

Supplement: Supplementary Figure S1 [file emi201757x1.docx]
