# Supplementary material for: Dengue burden in India: recent trends and importance of climatic parameters
Source: Emerg Microbes Infect. 2017 Aug 9;6(8):e70–. doi: 10.1038/emi.2017.57 (PMC5583666; doi:10.1038/emi.2017.57)
Supplement: Supplementary Table S1 [file emi201757x2.docx]

**Title:** Dengue burden in India: Recent trends and importance of climatic parameters

**Authors:** Srinivasa Rao Mutheneni, Andy Morse, Cyril Caminade, Suryanaryana Murty Upadhyayula

**Supplementary Table-1:** Correlation analysis between dengue incidence and EIP in different states of India.

| **State** | **r-Value** | **P- Value** |
| --- | --- | --- |
| A.& N. Islands | 0.1 | 0.31 |
| Andhra Pradesh | 0.16 | 0.47 |
| Arunachal Pradesh | -0.19 | 0.53 |
| Assam | -0.37 | 0.85 |
| Bihar | -0.3 | 0.75 |
| Chandigarh | -0.04 | 0.12 |
| Chhattisgarh | 0.37 | 0.85 |
| D.& N.Haveli | 0.26 | 0.68 |
| Daman & Diu | 0.22 | 0.6 |
| Delhi | -0.27 | 0.7 |
| Goa | -0.21 | 0.59 |
| Gujarat | 0.03 | 0.1 |
| Haryana | 0.12 | 0.34 |
| Himachal Pradesh | -0.08 | 0.24 |
| Jammu & Kashmir | -0.28 | 0.72 |
| Jharkhand | -0.01 | 0.04 |
| Karnataka | -0.09 | 0.27 |
| Kerala | 0.23 | 0.63 |
| Lakshadweep | NA | NA |
| Madhya Pradesh | -0.19 | 0.54 |
| Maharashtra | 0.16 | 0.45 |
| Manipur | 0.33 | 0.8 |
| Meghalaya | 0 | 0.92 |
| Mizoram | -0.05 | 0.16 |
| Nagaland | -0.2 | 0.55 |
| Odisha | 0.43 | 0.91 |
| Puducherry | 0.2 | 0.55 |
| Punjab | -0.08 | 0.25 |
| Rajasthan | 0.13 | 0.37 |
| Sikkim | -0.06 | 0.19 |
| Tamil Nadu | 0.07 | 0.21 |
| Tripura | -0.22 | 0.61 |
| Uttar Pradesh | -0.29 | 0.75 |
| Uttarakhand | -0.22 | 0.61 |
| West Bengal | -0.19 | 0.54 |
